# Minding One's Reach (To Eat): The Promise of Computer Mouse-Tracking to Study Self-Regulation of Eating

**DOI:** 10.3389/fnut.2018.00043

**Published:** 2018-05-22

**Authors:** Richard B. Lopez, Paul E. Stillman, Todd F. Heatherton, Jonathan B. Freeman

**Affiliations:** ^1^Department of Psychology, Rice University, Houston, TX, United States; ^2^Department of Psychology, Ohio State University, Columbus, OH, United States; ^3^Department of Psychological & Brain Sciences, Dartmouth College, Hanover, NH, United States; ^4^Department of Psychology, New York University, New York, NY, United States

**Keywords:** Self-regulation, eating behavior, mouse-tracking, decision-making, individual differences

## Abstract

In this review, we present the case for using computer mouse-tracking techniques to examine psychological processes that support (and hinder) self-regulation of eating. We first argue that computer mouse-tracking is suitable for studying the simultaneous engagement of—and dynamic interactions between—multiple perceptual and cognitive processes as they unfold and interact over a fine temporal scale (i.e., hundreds of milliseconds). Next, we review recent work that implemented mouse-tracking techniques by measuring mouse movements as participants chose between various food items (of varying nutritional content). Lastly, we propose next steps for future investigations to link behavioral features from mouse-tracking paradigms, corresponding neural correlates, and downstream eating behaviors.

Higher-order human cognition often comprises of multiple, interacting processes that ultimately impact downstream behavior. This is particularly true when people attempt to control their behavior in accordance with goals to maintain health and well-being. A prime example of this is the difficulty many individuals face when attempting to manage their food intake. Indeed, short-term impulses to eat are often in conflict with long-term health goals, and regulatory processes need to intervene and dynamically modulate or suppress those impulses so people can make healthier choices (1–3). And given the modern obesogenic environment (4, 5), coupled with obesity rates pushing 35–40% in the United States (6), it has become critical for people to exert control over non-homeostatic eating.

The fields of experimental psychology and cognitive neuroscience are well-suited to determine why, and under which circumstances, people fail to regulate their eating and make poor choices that undermine their health. Investigators in these fields have access to newly developed, freely available, and broadly accessible tools that better measure psychological processes and their neural correlates. One such tool is computer mouse-tracking, which continuously measures the position and velocity of hand movements while participants make forced-choice decisions—allowing for assessment of how multiple cognitive processes unfold and interact, in real time, to guide those decisions (7, 8).

In a typical mouse-tracking paradigm, participants make repeated forced-choice decisions by moving the cursor from the bottom-center of the screen to responses in either top corners of the screen (see Figure 1). These decisions are often either categorization judgments (e.g., categorizing a food as good or bad), or preference indications (electing one of two food options). Of interest is the hand-movement trajectory en route to the ultimate response. Certain factors may lead participants to tentatively consider and thus be partially drawn toward the opposite response (on the opposite side of the screen) (9). Beyond such attraction effects, other aspects of the hand trajectory, such as its stability or instability (10), the velocity and acceleration (11), and the angle of movement (12) are also informative. As such, the mouse-tracking paradigm breaks down a single choice into a continuous stream of cognitive output, revealing how tentative commitments to multiple potential responses coalesce into a single response over time [for review, (7, 13, 14)]. In these ways, the mouse-tracking technique is especially sensitive to the partial influences of multiple competing goals and biases that inevitably guide perceptual judgments and decisions.

**Figure 1:**
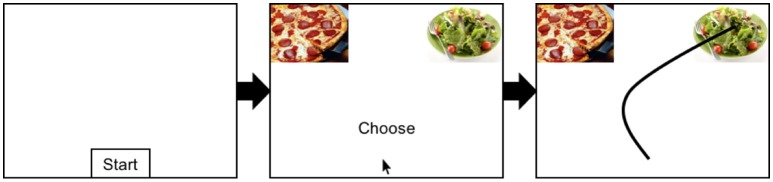
Schematic depiction of a typical mouse-tracking trial. First, participants click on a start button at the bottom-center of the screen to initiate the trial, then two options (e.g., high and low calorie food items) appear at the top-left and top-right corners of the screen, respectively. Participants then freely move the mouse toward the desired food.

Researchers across multiple labs have successfully developed programs of research based off of mouse-tracking methods in the domains of language [e.g., (15)], vision (16), and intertemporal choice (17). However, there have been comparatively fewer studies using mouse-tracking to gain a real-time understanding of how people arrive at their eating decisions. Notably, mouse-tracking may be uniquely suited to studying self-regulatory processes in the context of eating choices, as these choices often involve multiple conflicting goals (e.g., immediate consumption, maintain health, and/or outward appearance), that compete to drive choices. By tapping into the complex unfolding of such decisions, mouse-tracking may thus reveal the processes underlying eating choices in ways that traditional self-report metrics, which are prone to memory and other biases [e.g., (18)], cannot. Moreover, and as argued by some of first researchers to develop and validate mouse-tracking methods [e.g., see (19)], mouse-tracking has some methodological advantages over eye-tracking methods. Although eye-tracking can reliably index attention, including attentional processing of nutrition information in the context of eating decisions (20), eye-tracking patterns can be ballistic and discrete, while mouse-tracking measures motor output continuously and is therefore more sensitive to subtle attraction effects.

In this review, we present the case for using computer mouse-tracking to examine underlying psychological processes that support (and hinder) self-regulation of eating. First, we describe how computer mouse-tracking is uniquely suitable for studying the simultaneous engagement of—and dynamic interactions between—multiple perceptual and cognitive processes as they unfold and interact over a fine temporal scale (i.e., hundreds of milliseconds). Next, we review recent work that has used mouse-tracking to better understand food decisions—specifically, we review work that uses mouse-tracking to investigate (a) the relative conflict people experience when choosing healthy over unhealthy, (b) the timing with which information is processed, and (c) the strategies that people use to elect healthy over unhealthy food. Lastly, we lay out concrete next steps for future investigations to link behavioral features from mouse-tracking paradigms, corresponding neural correlates, and downstream eating behaviors.

## Measuring real-time decisions with computer mouse-tracking

Mouse-tracking takes advantage of recent research in cognitive psychology suggesting that motor output is continuously updated to reflect underlying cognitive processing (21, 22). Early work validating mouse-tracking demonstrated that cursor movements were an approximate reflection of the evolution of a categorization judgment or choice, whether making basic categorical judgments about animals (19), or more complex judgments about social stimuli (i.e., faces), some of which were sex-typical and others sex-atypical [i.e., faces with a mixture of masculine and feminine features; (23)]. This work showed, for instance, that mouse-tracking served as a sensitive measure of both the conflict present between two response options, as well as the temporal resolution of that conflict [for reviews, see (9, 13)]. Over the past decade, mouse tracking techniques have been applied to other disciplines, including social psychology, to validate various models of perception and cognition. For instance, Freeman and Ambady's (24) model tested predictions of dynamic (rather than sequential) influence of knowledge about racial stereotypes on the one hand, and visual features on the other, by showing that mouse-trajectories were jointly influenced by both types of information—even from the beginning of a categorization judgment (25). More closely related to the study of appetitive behaviors and eating decisions, dynamic models such as Gladwin et al.'s (26) Reinforcement/Reprocessing model of Reflectivity proposes that the relative time courses of impulsive and regulatory processes are critical to understand outcome behaviors with implications for health, including addictive behaviors (26).

## Applying mouse-tracking to the self-regulation of eating

In the domain of self-regulation, especially self-regulation of eating, conflicting and competing goals abound. Impulses to eat are often triggered by incidental exposure to appetitive cues (27–29) and readily motivate us to seek out and consume the desired food item. However, if we are motivated to control our eating, a separate set of regulatory goals may come online and intervene to curb the initial, activating impulse [(1, 2, 30)]. By continuously measuring motor output, mouse-tracking may help illuminate how these goals compete in real time to shape and drive ultimate eating decisions. This, in turn, may allow for a deeper understanding of how different self-regulatory strategies facilitate (or inhibit) healthy food choices—with the potential benefit of increasing people's awareness of how their goals and goal conflicts play into their eating choices. Further, unlike domains of categorization, motor movement towards depicted food items on a computer screen has close parallels to experiences individuals face in everyday life, especially when presented with multiple food items on a kitchen table or at a buffet. For instance, we may gravitate toward a piece of cake before grabbing an apple, indicative of successful control of an initial impulse. In another case, the desire for sweets might overwhelm completely, leading to a mad dash to a plate of cookies.

Although this may resonate with our intuitions of how we make eating decisions, researchers have employed mouse-tracking techniques to unobtrusively examine hand movements that guide and underlie food choice in laboratory settings. The richness of data offered by mouse-tracking allows researchers to investigate many complementary components of self-regulation. Although the precise goals vary by research program, mouse tracking in the eating domain has to-date been used to address three distinct goals: to get a more sensitive metric of conflict when choosing between healthy and unhealthy foods, to understand the temporal dynamics of eating decisions, and to identify the nature in which these decisions evolve. We note at the outset that many of these experiments use food items that have been selected based on pretests of how healthy or tasty participants believe them to be, and/or caloric content [by volume; see (31)]. This is distinct, however, from which foods are *actually* healthy and nutritious, as people encounter them in real world settings, especially restaurants. For instance, although naïve participants typically believe salad to be far healthier than pizza, modern techniques of preparing these foods may dramatically reduce the healthiness of the salad, to the point where it may be equivalent—as far as fat and/or overall caloric content—with pizza. While this is ideal for investigating underlying self-regulatory processes, it is important to note this will sometimes limit the direct applicability of this research to real-world decisions. However, future work can develop and validate additional food stimuli banks that more closely reflect the kinds of food items and choices that people are exposed to in their daily lives, especially prepared foods when eating out. Furthermore, and as mentioned in the discussion section below, a limitation of current mouse-tracking studies is that food choice outcomes measured in the lab should be substantiated by corresponding real world eating patterns.

### Conflict in eating choices

One strength of mouse-tracking is it allows researchers to more directly quantify the response conflict given decision elicits by measuring the extent to which participants are drawn towards the unchosen option (e.g., the relative amount the mouse is pulled towards the pizza when choosing the salad in Figure 1).

Conflict is often assumed to be integral in decisions between healthy vs. unhealthy foods, and recently researchers have begun to use mouse-tracking to probe this conflict. In an initial study, Ha et al. (32) explored decision-making and self-regulation of eating in pre/early adolescence [ages 8–13; (32)]. In this study, children's computer mouse movements were recorded as the children freely chose between healthy (e.g., vegetables and beans) and unhealthy (e.g., sweet desserts, fried foods) food items. Generally, children preferred unhealthy food items over healthy ones, and showed marked difficulty to avoid tempting (unhealthy) foods—as indicated by a relatively larger area under the curve (AUC) when they decided not to choose *unhealthy* items, as compared to not choosing healthy items [see Figure 2 in (32)]. In mouse-tracking studies, AUC is a common metric to estimate response conflict and is calculated by taking the difference between the ideal (i.e., straight point-to-point) trajectory and the observed trajectory, with larger AUC indexing more competition between the two response options. In the case of Ha et al.s' (32) study, larger AUC may be indicative of a relatively stronger desire to eat the unhealthy food, and a possible indicator of children's inability to regulate such impulses.

Consistent with this, Stillman et al. (33) investigated food choices^1^ in adults, and found that those with higher self-reported self-control had more direct mouse trajectories when choosing healthy over unhealthy foods, suggesting that the relative strength of goals and temptations is related to individuals' self-control. Further, these researchers found that AUC when choosing healthy over unhealthy foods was marginally related to a whether participants chose an apple over a candy bar at the conclusion of the study, such that the more participants were drawn to the unhealthy food while choosing the healthy food, the less likely they were to elect the apple. Together, these studies suggest that conflict can be a predictive component of eating decisions, and mouse-tracking can sensitively measure this conflict.

Whereas the above studies focused on food choices, other researchers have employed mouse tracking to study evaluations of food—in other words, whether people view different foods as positive or negative. Schneider et al. (34) assessed participants' attitudes as they related to different classes of stimuli (including food), and reasoned that unhealthy foods and alcohol were more likely to elicit ambivalence than other (healthy) foods (34). They found a main effect of ambivalence on mouse trajectories, with a greater “pull” toward ambivalent attitude objects (e.g., beer, hamburger), which likely elicited self-regulatory conflict [e.g., see (35)], compared with non-ambivalent attitude objects (e.g., orange juice, apple). Additionally, the time at which “pull” was greatest generally occurred later (at about 868 ms) for ambivalent stimuli than non-ambivalent stimuli (674 ms), meaning that ambivalence caused longer response conflict, potentially due to co-activation of competing features of the stimulus (e.g., hedonic qualities *and* regulatory goals elicited by the ambivalent/unhealthy food items).

A study in a similar vein by Gillebaart et al. (36) focused on trait self-control (37) as a key moderating variable in people's positive and negative evaluations of healthy and unhealthy foods (36). Critically, those high in trait self-control reached the maximum deviation in their mouse trajectories sooner on trials in which they overcame response conflict—by ultimately classifying healthy foods as positive and unhealthy foods as negative—than those with lower trait self-control (36). Taken together, both Schneider and Gillebaart's studies highlight the role of valence as an important dimension along which people process and respond to food items, and how it can alter the temporal dynamics of decisions to choose between healthy and unhealthy options.

### Temporal dynamics of attribute integration

Beyond investigating conflict, researchers can use the fine-grained temporal information to investigate when in the time-stream a given attribute—in this case, healthiness and tastiness—influences participants' mouse movements. In the first such study, Sullivan et al. (12) showed adult participants pairs of food items that varied along dimensions of tastiness (i.e., “how tasty is the food”) and healthfulness (i.e., “how healthy is the food”), and measured mouse movements as participants freely chose between different pairs of foods (12). Across decisions, they then calculated *when* in the time-course health and taste information began to significantly influence mouse movements. Critically, they observed a main effect whereby tastiness attributes of food items were processed more quickly (about 195 ms earlier) than healthfulness attributes—as indicated by altered spatial patterns in mouse trajectories. Lim et al. (38) extended these findings, replicating the effect that tastiness was more quickly incorporated than healthfulness, but that this comparative advantage was reduced when presenting calorie information alongside the foods (38). These findings suggest that on a relatively fine time scale, people can readily process certain features of appetitive cues, and differences in the temporal dynamics of how features become incorporated into a motor response (i.e., an approach or reaching behavior) may impact downstream decisions to eat healthily (or poorly).

### Nature of decision evolution

Finally researchers have begun to use mouse-tracking to better understand *how* people successfully chose healthy over unhealthy foods. For instance, certain frameworks of eating decisions emphasize impulse inhibition [e.g., (2)], in which an initial impulse is automatically activated in response to a tempting food, and then—given adequate motivation and ability—are inhibited by slower, more controlled processes. Dynamic accounts, however, emphasize the many automatic processes that can (under certain conditions) automatically activate in response to a temptation [e.g., (39–41)], and then dynamically compete with the temptation. Importantly, these different accounts predict different trajectory evolution signatures. The first (impulse inhibition) account predicts a direct trajectory towards the unhealthy food, followed by a large midflight correction back towards the goal, thus yielding trajectories that appear abrupt. The second (dynamic competition) account, on the other hand, predicts ongoing competition between the temptation and goal, and thus predicts trajectories that are graded rather than abrupt. When testing between these accounts, Stillman et al. (33) found that the majority of decisions (i.e., 74%) in which participants elected healthy over unhealthy appeared to evolve in a graded fashion, thus more consistent with the dynamic competition account. Notably, however, a non-trivial number of decisions (roughly 26% across studies) did evoke trajectories consistent with impulse-inhibition, suggesting that both dynamic competition and impulse inhibition can occur (see Figure 1 in (33) showing a schematic depiction of mouse trajectories consistent with dual-systems and dynamic competition models, respectively). In this way, mouse-tracking can be used not only to help test models of healthy eating decisions, but also to shed light on the specific strategies people employ to avoid tempting foods.

## Conclusion and future directions

Here, we have reviewed recent studies that have applied computer mouse-tracking techniques to the study of decision-making processes in the context of self-regulation of eating behaviors. Specifically, researchers have used these techniques to investigate conflict, time-courses, and strategies used as people made decisions between healthy and unhealthy foods in real time. Although this work is in its relative early days, some conclusions are warranted at this point. First, across the studies we have discussed, mouse trajectories seemed to reveal subtle and potentially unconscious biases in how people process and respond to tempting food cues. Although different dimensions of the cues were operationalized, there was frequently some level of response conflict, as indicated by mouse-trajectory measures computed from participants' hand trajectories.

Future work might be able to make more specific inferences about which cognitive processes are recruited as people attempt to regulate their eating, by applying various computational models to mouse-tracking data and observing which parameters track with the timing and magnitude of trajectory measures (10). For example, either by testing naturally occurring differences or by directly manipulating variables and contexts (e.g., hunger, stress, dieting goals), researchers can compare more traditional, dual-process models of self-control [e.g., (2)] with newer models that posit multiples inputs into a general valuation process, which then would guide subsequent behavior (42).

Additionally, with noninvasive brain imaging (i.e., fMRI) researchers can assess brain responses to food cues, even relatively spontaneous and incidental activity [e.g., (43, 44)], and test whether individual differences in neural food cue reactivity (e.g., increased responsivity in brain reward regions, such as ventral striatum or orbitofrontal cortex) are associated with altered mouse trajectories as people make decisions to eat healthy or unhealthy foods [for a similar approach in social categorization research, see (45)]. In this way, one could determine whether potential differences in temporal dynamics and decision biases—as measured by mouse trajectories—may reveal individual differences in processing of food cues and potential risk for subsequent self-control failure.

One notable limitation of the studies we have reviewed is that the eating decisions participants made were often hypothetical and took place within a laboratory context. It will be important for future research to examine the effects of the “pull” toward competing food options on real-world eating behaviors and decisions *outside* the laboratory. For example, a natural extension of this work would be to record 3D hand movements en route to real food options that participants could select and consume. Ideally, hand movements would be measured *without* participants explicit knowledge that their eating decisions were being assessed. For example, a food choice task could be embedded or interleaved with other choice tasks (i.e., choosing objects with relatively little appetitive value and neutral valence). One might also consider incorporating experience sampling protocols delivered via participants' smartphones (46) into such study designs, which would enable researchers to explore relationships between mouse or hand trajectory patterns, daily eating decisions, and general patterns of consumption over time. Indeed, this kind of study design would be crucial in establishing ecological validity of mouse-tracking effects observed in laboratory settings—namely whether choice preferences as indicated by a mouse-tracking task reliably tracks with people's actual eating decisions in daily life.

Another caveat to the mouse-tracking studies reviewed here is that most of them have not taken into account important factors and individual difference measures that undoubtedly impact eating decisions. First, future mouse-tracking studies would benefit from implementing between-group designs, such as comparing trajectories during food choice trials between populations who variably regulate their eating (e.g., chronic dieters vs. non-dieters). The fact that many psychology studies—including some discussed here—recruit from the undergraduate aged population also constrains inference and limits generalizability of the observed findings. Moreover, key individual difference factors, such as people's idiosyncratic food preferences, habitual eating patterns, and beliefs about body weight, and nutritional literacy would be critical to incorporate into mouse-tracking studies. This can be done either by controlling for these factors to examine main effects, or to investigate unique, specific contributions of these factors to decision-making processes that drive eating behaviors.

To conclude, we believe that computer mouse-tracking, in close conjunction with brain imaging and other assessments of personality and behavior, holds much promise to unravel complex decision-making processes and frequent self-control dilemmas many face in our modern world.

## Author contributions

RL and PS performed the literature review that informed the organization and sections of the mini-review; RL wrote the initial draft of the manuscript; TH and JH made subsequent edits; PS helped RL make final edits.

### Conflict of interest statement

The authors declare that the research was conducted in the absence of any commercial or financial relationships that could be construed as a potential conflict of interest. The reviewer MH and handling Editor declared their shared affiliation.

## Footnotes

^1^In these studies, participants' choices were restricted, such that participants were instructed to always choose the healthy over unhealthy food. However, subsequent studies in other domains (intertemporal choices) found similar patterns with unrestricted choices.
